# Normative ocular biometric profile of emmetropic Nepalese children: a hospital-based cross-sectional study

**DOI:** 10.1186/s12886-026-05405-4

**Published:** 2026-10-01

**Authors:** Archana Sharma, Madhu Thapa, Sanjeev Bhattarai, Harshita Thapa, Pratima Lama Ghising, Sarmila Acharya, Gauri Shankar Shrestha

**Affiliations:** Department of Ophthalmology, B.P. Koirala Lions Centre for Ophthalmic Studies, Maharajgunj Medical Campus, Institute of Medicine, Tribhuvan University, Kathmandu, Nepal

**Keywords:** Average central corneal power (ACCP), Corneal eccentricity index (CEI), Fourier index, Nepalese children, Optical biometer

## Abstract

**Purpose:**

To evaluate ocular biometric parameters and corneal topographical parameters in emmetropic Nepalese children using swept-source optical biometer.

**Methods:**

This cross-sectional study included 190 emmetropic Nepalese children aged 6–18 years. Ocular biometric parameters (Corneal radius of curvature, Axial length, Anterior chamber depth, Lens thickness, Pachymetry, Pupil diameter, White to white diameter) and corneal topographical parameters (Corneal eccentricity index, Average central corneal power, Fourier index) were measured using OA-2000 optical biometer. Data were analyzed using SPSS version 27. Correlation between key ocular biometric parameters were analyzed using Pearson’s correlation.

**Results:**

The study included 97 males (51.1%) and 93 females (48.9%), with a mean age of 11.99 ± 3.40 years. Mean flat and steep corneal radius of curvature were 7.91 ± 0.24 mm and 7.78 ± 0.24 mm, respectively. Mean axial length was 23.03 ± 0.67 mm, anterior chamber depth 3.55 ± 0.26 mm, lens thickness 3.56 ± 0.20 mm, pachymetry 527.4 ± 32.50 μm, pupil diameter 5.93 ± 0.69 mm, white-to-white diameter 12.02 ± 0.39 mm, and axial length to corneal radius ratio 2.93 ± 0.07. Males demonstrated significantly larger ocular biometric values than females for all parameters except lens thickness (*p* < 0.05). Axial length was positively correlated with corneal radius of curvature (*r* = 0.70, *p* < 0.001) and anterior chamber depth (*r* = 0.40, *p* < 0.001).

**Conclusions:**

Emmetropic Nepalese children exhibit significant gender-related differences for most of the ocular biometric parameters; however, the magnitude of these differences varied, and clinically meaningful differences were primarily noted for axial length and white-to-white diameter. These findings establish normative biometric and topographical data for the Nepalese pediatric population.

## Background

Emmetropization is a coordinated process of ocular growth during childhood in which changes in axial length, corneal curvature, anterior chamber depth, and crystalline lens power and geometry occur in a balanced manner to maintain the eye’s optical focus near the retinal plane and minimize refractive error. As the eye grows, axial elongation tends to increase the eye’s myopic refractive tendency, while reductions in corneal and crystalline lens power provide compensatory changes that help maintain emmetropia [1, 2]. Thus, emmetropization reflects the coordinated adjustment of ocular dimensions and optical power rather than simple enlargement of the eye. When these structural and optical changes become disproportionate, refractive error may develop including myopia and hyperopia [3]. Accurate measurement of pediatric ocular biometric parameters is essential for evaluating refractive development, monitoring physiological growth and detecting early deviations. Axial length, in particular, serves as a critical biomarker; values exceeding age-specific norms signal increased risk of high myopia.

Globally, normative pediatric ocular biometric data remain sparse, with limited reports from South Asian populations [4–6]. In Nepal, existing evidence derives largely from school-based studies using ultrasound contact biometry [6]. This method, however, measures axial length only to the inner limiting membrane (not the retinal pigment epithelium) and underestimates anterior chamber depth due to corneal indentation [7]. These limitations have prompted adoption of non-contact optical biometers, which offer automated, multi-parameter assessment with high repeatability, particularly in children.

The OA-2000 (Tomey Corporation, Nagoya, Japan) is a non-contact swept-source optical biometer incorporating Placido-disc corneal topography and integrated 3D eye tracking technology. It utilizes a 1060 nm scanning laser to obtain precise measurements of axial length, anterior chamber depth, lens thickness, pachymetry, pupil diameter, white-to-white diameter, and derived indices including corneal eccentricity index (CEI), average central corneal power (ACCP), and Fourier indices (spherical power, regular astigmatism, asymmetry, higher-order aberrations) [8]. 

Establishing normative ocular biometric data requires assessment of emmetropic eyes, as they represent physiological ocular growth without the confounding influence of refractive error–related structural alterations. Therefore, this hospital-based study aimed to establish a normative database of ocular biometric parameters in emmetropic Nepalese children using non-contact optical biometer. The hospital setting enabled standardized examination conditions and access to advanced imaging technology. The resulting reference values are expected to enhance understanding of eye growth patterns in Nepalese children and provide a valuable resource for future research and clinical application, including myopia control strategies and pediatric intraocular lens power calculations.

## Methods

### Study design and participants

This cross- sectional study included 190 emmetropic Nepalese children aged 6–18 years who attended the ophthalmic outpatient clinics during the study period. The minimum sample size was determined to be 172 using Cochran’s formula for a finite population: 95% confidence level, 5% margin of error, population proportion of 0.5, and a population size of 310 (based on half-yearly outpatient records of healthy children at BPKLCOS, 2024–2025). To account for a 10% non-probability sampling error, the final sample was increased to 190 to ensure adequate representation and statistical power. Participants were recruited consecutively following eligibility screening to minimize selection bias. The broad age range was selected to capture key stages of ocular growth from early childhood through late adolescence.

Ethical approval for the study was obtained from the Institutional Review Committee of the Institute of Medicine, Tribhuvan University (Protocol no: 300/081/082). Written informed consent was obtained from the parents or legal guardians of all participants, and assent was obtained from children when appropriate, depending on age and understanding.

### Ophthalmic examination and refraction

All participants underwent a comprehensive ophthalmic examination. Distance visual acuity was assessed monocularly at 6 m using an internally illuminated Snellen’s chart. Objective refraction was measured using autorefractometer (KR-800; Topcon Corporation, Nagoya, Japan), and the spherical equivalent refraction (SER) was calculated as sphere + ½ cylinder.

Cycloplegic refraction was subsequently performed to determine the true refractive status which was induced using two cycles of 1% cyclopentolate hydrochloride followed by one cycle of 0.5% tropicamide in each eye, administered at 10-minute intervals. Punctal occlusion was performed for 1–2 min after each instillation to minimize systemic absorption. Cycloplegia was assessed clinically 30 min after the final instillation based on the absence of the pupillary light reflex and diminished near visual acuity [9]. Although the emmetropia criterion varies across the literature, emmetropia was operationally defined as SER between + 0.75 D and − 0.75 D for the purpose of this study, allowing inclusion of physiologically emmetropic eyes while accounting for minor measurement variability inherent in pediatric refraction [10]. 

### Inclusion and exclusion criteria

Only eyes meeting the predefined Emmetropia criteria (SER between ± 0.75 D) were included. Exclusion criteria comprised significant refractive errors beyond this range, manifest strabismus, nystagmus, previous ocular morbidity or surgery, and systemic or neurodevelopmental conditions that could affect ocular growth, fixation, or measurement reliability.

### OA-2000 biometry and corneal topography protocol

**Fig. 1:**
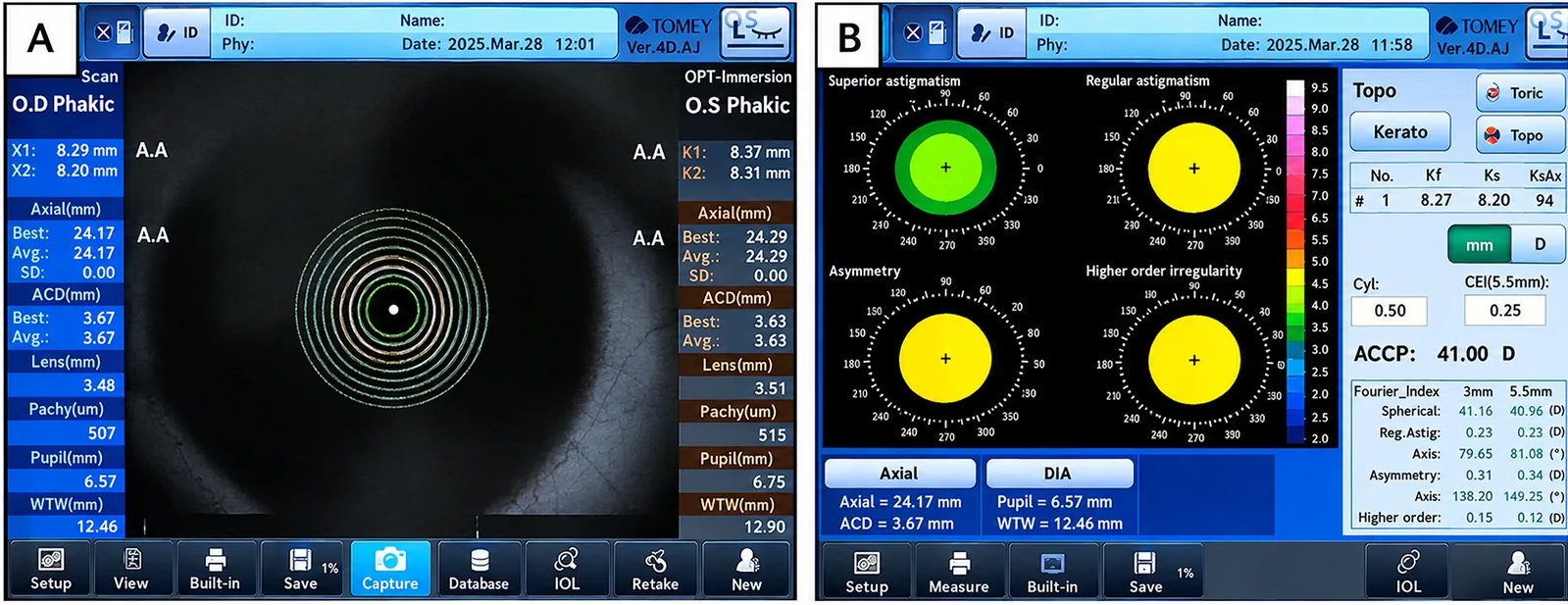
Representative OA-2000 outputs from both eyes of a 16-year-old emmetropic Nepalese child, demonstrating acquisition of axial length, anterior segment biometry, and Placido-disc–based corneal topography. The figure highlights bilateral measurement capability, fixation alignment, and integrated biometric and corneal data capture using the OA-2000 system

Ocular biometric and corneal topographical parameters were measured using the OA-2000 optical biometer (Tomey Corporation, Nagoya Japan), which combines swept- source optical biometry with Placido-disc-based corneal topography (Fig. 1). All measurements were performed by a single trained optometrist in a standardized examination room under consistent illumination conditions to minimize environmental variability.

Participants were instructed to fixate on the internal target of the instrument, and measurements were obtained only when stable fixation and proper alignment were achieved. In younger children, verbal encouragement and rest periods were provided as needed to optimize cooperation. Each biometric measurement was repeated at least three times per eye, and only scans with acceptable signal quality and alignment, as indicated by the device’s internal quality indicators, were retained. The mean of the three highest-quality readings was used for analysis.

Corneal curvature was measured using a Placido-disk topography over a 5.5 mm corneal zone, comprising nine concentric rings with 256 reference points as shown in Fig. 1. Flat (K1) and steep (K2) keratometry values were derived from two principal meridians oriented 90° apart, using a standardized refractive index of 1.3375, and evaluated at 2.5 mm diameter for comparability with existing literature.

Axial length was measured from the anterior tear film to the retinal pigment epithelium using swept-source technology, providing high repeatability and minimizing operator-dependent error [11]. Anterior chamber depth was measured from the anterior corneal epithelium to the anterior lens surface, without corneal indentation. Lens thickness was measured as the distance between the anterior and posterior lens surfaces. White-to-white (WTW) diameter was calculated as the horizontal limbus-to-limbus corneal diameter using automated edge detection [12]. 

Derived biometric indices were calculated to support interpretation of ocular growth patterns. The axial length-to-corneal radius (AXL/CR) ratio was calculated by dividing axial length by the mean corneal radius derived from K1 and K2. Average central corneal power (ACCP) was calculated by averaging refractive powers from the central Placido rings within the central 3.0 mm zone. Corneal eccentricity index (CEI) was derived over the 5.5 mm zone to describe corneal asphericity, where positive values indicate a prolate cornea, zero indicates a spherical cornea and negative values indicate an oblate cornea. Fourier analysis was used to decompose corneal power into spherical, regular astigmatism, asymmetry, and higher-order irregularity components, allowing a detailed assessment of corneal shape characteristics.

All OA-2000 measurements were obtained prior to cycloplegia to avoid pharmacologically induced changes in anterior chamber depth and lens thickness. Cycloplegic refraction was subsequently performed for refractive classification only.

### Statistical analysis

Collected data were checked, reviewed and organized for completeness and accuracy prior to analysis. Statistical analyses were performed using SPSS version 27 (IBM Corp., Armonk, N.Y., USA). Descriptive statistical measures were used to summarize demographic and ocular biometric parameters. Continuous variables were assessed for normality using the Shapiro-Wilk test. Normally distributed data are presented as mean ± standard deviation (SD) with corresponding 95% confidence intervals (CI), while non-normally distributed variables are reported as median with interquartile range (IQR).

To maintain statistical independence, biometric data from one eye per participant were included in the final analysis, with the study eye selected randomly when both eyes met inclusion criteria. Inter-eye comparisons were performed using paired t-tests for normally distributed variables and Wilcoxon signed-rank tests for non-normally distributed variables.

Comparison of ocular biometric parameters between males and females were performed using independent t-tests for normally distributed data, with homogeneity of variance assessed using Levene’s test. When the assumption of equal variance was violated, Welch’s t-test was applied. For non-normally distributed variables, the Mann–Whitney U test was used.

Participants were categorized into four age groups (6–9, 10–12, 13–15, and ≥ 16 years) to facilitate the assessment of age-related differences in ocular biometric parameters across childhood and adolescence. These age categories were selected as pragmatic analytical strata and were not intended to represent discrete biological stages of emmetropization. Differences in ocular biometric parameters across age groups and between genders were evaluated using two-way analysis of variance (ANOVA), with age group and gender as fixed factors. Assumptions of normality and homogeneity of variances were verified prior to analysis. Interaction effects between age group and gender were examined, and where significant main or interaction effects were identified, post-hoc pairwise comparisons with Tukey HSD were performed. Clustered box-and-whisker plots were used to visually represent age- and gender-related variations, with median values indicated.

Pearson’s correlation coefficient (r) was used to assess linear relationships between axial length and corneal radius of curvature, and between axial length and anterior chamber depth, for normally distributed variables. Scatter plots were examined to confirm linearity and identify potential outliers. When normality or linearity assumptions were not met, Spearman’s rank correlation coefficient was applied. All statistical tests were two-tailed, and a p-value < 0.05 was considered statistically significant.

## Results

### Demographic characteristics

A total of 190 emmetropic Nepalese children (mean SER: 0.02 ± 0.41 D) aged 6–18 years (mean age ± SD: 11.99 ± 3.4 years; range: 6–18 years) were enrolled in the study. Participants were categorized into four age groups representing early childhood (6–9 years), middle childhood (10–12 years), early adolescence (13–15 years), and late adolescence (≥ 16 years) where the participants were clustered in the middle childhood, while 16-years and above group constituted a smaller proportion of the sample as illustrated in Table 1.

**Table 1:** Demographic characteristics of study participants

Variables	*N* (%)
**Gender**	
Male	97 (51.1%)
Female	93 (48.9%)
**Age group**	
6–9 years	49 (25.8%)
10–12 years	57 (30.0%)
13–15 years	49 (25.8%)
16 years and above	35 (18.4%)
**Age (Mean ± Std. Dev)**	**11.99 ± 3.4 years**

### Bilateral measurements of ocular biometric and corneal topographical parameters

The distribution of ocular biometric parameters and corneal topographical parameters of right and left eye among emmetropic Nepalese children (Table 2).

Most ocular biometric parameters showed no statistically significant differences between right and left eye for flat corneal radius of curvature, steep corneal radius of curvature, axial length, anterior chamber depth, lens thickness, pupil diameter, and white-to-white corneal diameter (all p *>* 0.05), except pachymetry with small mean interocular difference of 2 μm (*p* < 0.05) indicating high interocular symmetry - only the right eye data for ocular biometric parameters were considered for further analysis.

In contrast, statistically significant interocular differences were observed for most of the corneal topographical parameters (*p* < 0.05) except average central corneal power and spherical component in 3 mm zone (*p* > 0.05). These topographical parameters were reported only as normative data of both eyes with no further analysis.

**Table 2:** Distribution of ocular biometric and corneal topographical parameters of both eyes among emmetropic Nepalese children

	Right Eye		Left Eye		
	Mean ± Std. Deviation	95% CI	Mean ± Std. Deviation	95% CI	*p*-value
Ocular Biometric Parameters					
Flat corneal radius of curvature (K1) in mm	7.91 ± 0.24	7.88–7.94	7.91 ± 0.24	7.88–7.95	0.62
Steep corneal radius of curvature (K2) in mm	7.78 ± 0.24	7.75–7.81	7.77 ± 0.24	7.74–7.80	0.16
Axial length (AXL) in mm	23.03 ± 0.67	22.93–23.03	23.02 ± 0.67	22.93–23.12	0.49
Anterior chamber depth (ACD) in mm	3.55 ± 0.26	3.51–3.59	3.55 ± 0.26	3.52–3.59	0.51
Lens thickness (LT) in mm	3.56 ± 0.20	3.53–3.59	3.55 ± 0.21	3.52–3.58	0.74
Pachymetry (Pachy) in µm	527 ± 32.50	523–532	529 ± 32.41	525–533	< 0.00
Pupil Diameter (PD) in mm	5.93 ± 0.69	5.84–6.03	5.97 ± 0.73	5.86–6.08	0.32
White-to-white diameter (WTW) in mm	12.02 ± 0.39	11.96–12.08	12.02 ± 0.39	11.97–12.08	0.62
AXL/CR	2.93 ± 0.07	2.92–2.94	2.93 ± 0.07	2.92–2.94	0.92
SER	0.02 ± 0.41	-0.03 -0.08	0.04 ± 0.42	-0.01 -0.10	0.34
Corneal Topographical Parameters					
Corneal eccentricity index in 5.5 mm corneal zone (CEI)	0.39 ± 0.19	0.37–0.42	0.35 ± 0.23	0.32–0.39	0.00
Average central corneal power in 3 mm corneal zone (ACCP) (D)	43.02 ± 1.31	42.83–43.21	43.02 ± 1.28	42.83–43.20	1.00
Spherical component in 3 mm corneal zone (D)	43.31 ± 1.31	43.12–43.50	43.30 ± 1.28	43.12–43.49	0.63
Regular astigmatism in 3 mm corneal zone (D)	0.38 ± 0.19	0.35–0.41	0.42 ± 0.19	0.39–0.45	0.00
Asymmetry in 3 mm corneal zone (D)	0.44 ± 0.16	0.42–0.46	0.48 ± 0.16	0.45–0.50	0.00
Higher order aberration in 3 mm corneal zone (D)	0.18 ± 0.04	0.18–0.19	0.20 ± 0.05	0.19–0.20	0.02
Higher order aberration in 5.5 mm corneal zone (D)	0.15 ± 0.03	0.15–0.16	0.16 ± 0.04	0.16–0.17	< 0.00

Abbreviations: CI: confidence interval (95% for mean); D: diopters; CR: corneal radius of curvature calculated by average of sum of flat and steep corneal radius of curvature. Note: All parameters were normally distributed and analyzed using paired-t test. *Statistically significant difference (*p* < 0.05). All tests were two-tailed

Values shown as mean ± standard deviation (95% confidence interval)

### Comparison of gender based ocular biometric parameters- right eye measurements only

Gender-based comparisons demonstrated statistically significant differences for all ocular biometric parameters except pupil diameter and pachymetry (*p* < 0.05) (Table 3). However, larger differences were observed for axial length (approximately 0.41 mm; Cohen’s d = 0.63) and white-to-white diameter (approximately 0.19 mm; Cohen’s d = 0.50), whereas differences in K1, K2, ACD, and lens thickness were small, reflecting larger anterior segment dimensions in males. Lens thickness exhibited a small negative effect size (d = − 0.31), indicating slightly greater values in females. In contrast, pachymetry and pupil diameter did not differ significantly between genders and showed negligible to small effect sizes. Despite statistical significance for majority of ocular biometric parameters, clinically meaningful gender differences were primarily limited only to axial length and white-to-white diameter.

**Table 3:** Comparison of gender based ocular biometric parameters among emmetropic Nepalese children

Ocular Biometric Parameters	Gender	Mean ± Std. Deviation	Effect Size (Cohen’s d)	*p*-values*
Flat corneal radius of curvature (K1) in mm	Male	7.95 ± 0.24	0.30	0.04*
	Female	7.88 ± 0.21		
Steep corneal radius of curvature (K2) in mm	Male	7.82 ± 0.24	0.37	0.01*
	Female	7.74 ± 0.21		
Axial length (AXL) in mm	Male	23.23 ± 0.65	0.63	< 0.01*
	Female	22.82 ± 0.62		
Anterior chamber depth (ACD) in mm	Male	3.60 ± 0.26	0.41	0.01*
	Female	3.49 ± 0.25		
Lens Thickness (LT) in mm	Male	3.53 ± 0.20	-0.31	0.03*
	Female	3.59 ± 0.19		
Pachymetry in µm	Male	528 ± 32.76	0.03	0.82
	Female	526 ± 32.39		
Pupil diameter (PD) in mm	Male	6.01 ± 0.71	0.22	0.13
	Female	5.86 ± 0.67		
White-to-white diameter (WTW) in mm	Male	12.11 ± 0.38	0.50	< 0.01*
	Female	11.92 ± 0.38		

Note: All parameters were normally distributed and analyzed using Independent-t test. *Statistically significant difference (*p* < 0.05). All tests were two-tailed

### Variations of ocular biometric parameters with respect to age group and gender

**Fig. 2:**
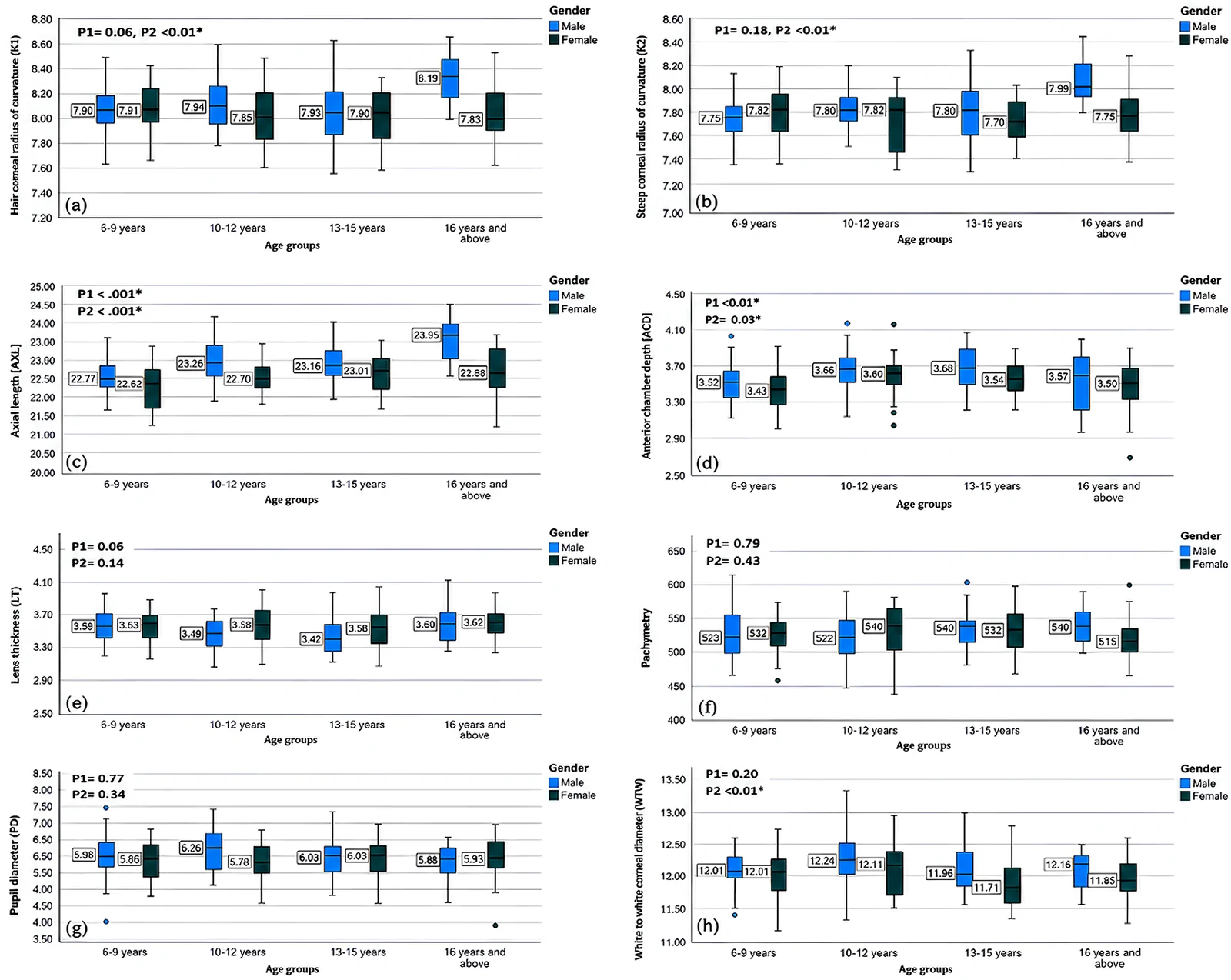
Reported data depicts the median with box-and-whisker plots showing variations of ocular biometric parameters (**a**, **b**) Flat and Steep corneal radius of curvature (K1, K2), (**c**) Axial length (AXL), (**d**) Anterior chamber depth (ACD), (**e**) Lens thickness (LT), (**f**) Pachymetry (PACHY), (**g**) Pupil diameter (PD), (**h**) White- to- white diameter (WTW) for the respective age groups and gender with p-values. The box represents the upper and lower quartiles and the whisker indicates 1.5 times the interquartile range. P1 = p-values generated using Two-way ANOVA for age groups and P2 for gender for all ocular biometric parameters

The variations of ocular biometric parameters with respect to age group and gender with their median values are demonstrated using clustered box plot which is shown in Fig. 2.

A two-way ANOVA was performed to evaluate the effects of age group, gender, and their interaction (age group × gender) on ocular biometric parameters. A statistically significant main effect of age group was observed on axial length (F _(3,182)_ = 8.60, p *<* 0.001) and anterior chamber depth (F _(3,182)_ = 4.13, *p* < 0.01), while no statistically significant main effect of age group was observed on flat and steep corneal radius of curvature, lens thickness, pachymetry, pupil diameter and white-to-white corneal diameter (all *p* > 0.05).

Similarly, statistically significant main effect of gender was observed on flat corneal radius of curvature (F _(1,182)_ = 7.92, p *<* 0.01), steep corneal radius of curvature (F _(1,182)_ = 11.36, *p* < 0.01), axial length (F _(1,182)_ = 25.13, p *<* 0.001), anterior chamber depth (F _(1,182)_ = 4.86, *p* = 0.03) and white-to-white corneal diameter (F _(1,182)_ = 7.89, *p* < 0.01). However, the age group × gender interaction was significant for flat corneal radius of curvature (F _(3,182)_ = 2.85, *p* = 0.04) and steep corneal radius of curvature (F _(3,182)_ = 3.51, *p* = 0.02), indicating that gender differences in corneal curvature varied across age groups. No significant age group × gender interactions were observed for axial length, anterior chamber depth, lens thickness, pachymetry, pupil diameter, or white-to-white corneal diameter indicating that gender differences for these biometric parameters were consistent across age groups (all *p* > 0.05).

Post hoc Tukey HSD analysis showed no significant age-group differences in flat and steep corneal radius of curvature, lens thickness, pachymetry and pupil diameter between any age groups (*p* > 0.05). Axial length was significantly shorter in the 6–9-year age group compared with all older groups (10–12, 13–15, and ≥ 16-year groups, *p* < 0.01), with no significant differences among the older age groups (*p* > 0.05), demonstrated in Fig. 2(c). Anterior chamber depth was significantly shallower in the 6–9-year group than in the 10–12- and 13–15-year groups (*p* < 0.05), while the ≥ 16-year group also had significantly shallower ACD than the 10–12- and 13–15-year groups (*p* < 0.05) as shown in Fig. 2 (d). No significant difference was observed between the 6–9- and ≥ 16-year groups or between the 10–12- and 13–15-year groups (*p* > 0.05). White-to-white corneal diameter showed a significant difference only between the 10–12- and 13–15-year age groups (*p* = 0.05), with no significant differences among other age groups as depicted in Fig. 2(h).

### Correlation of axial length with corneal radius of curvature and anterior chamber depth

**Fig. 3:**
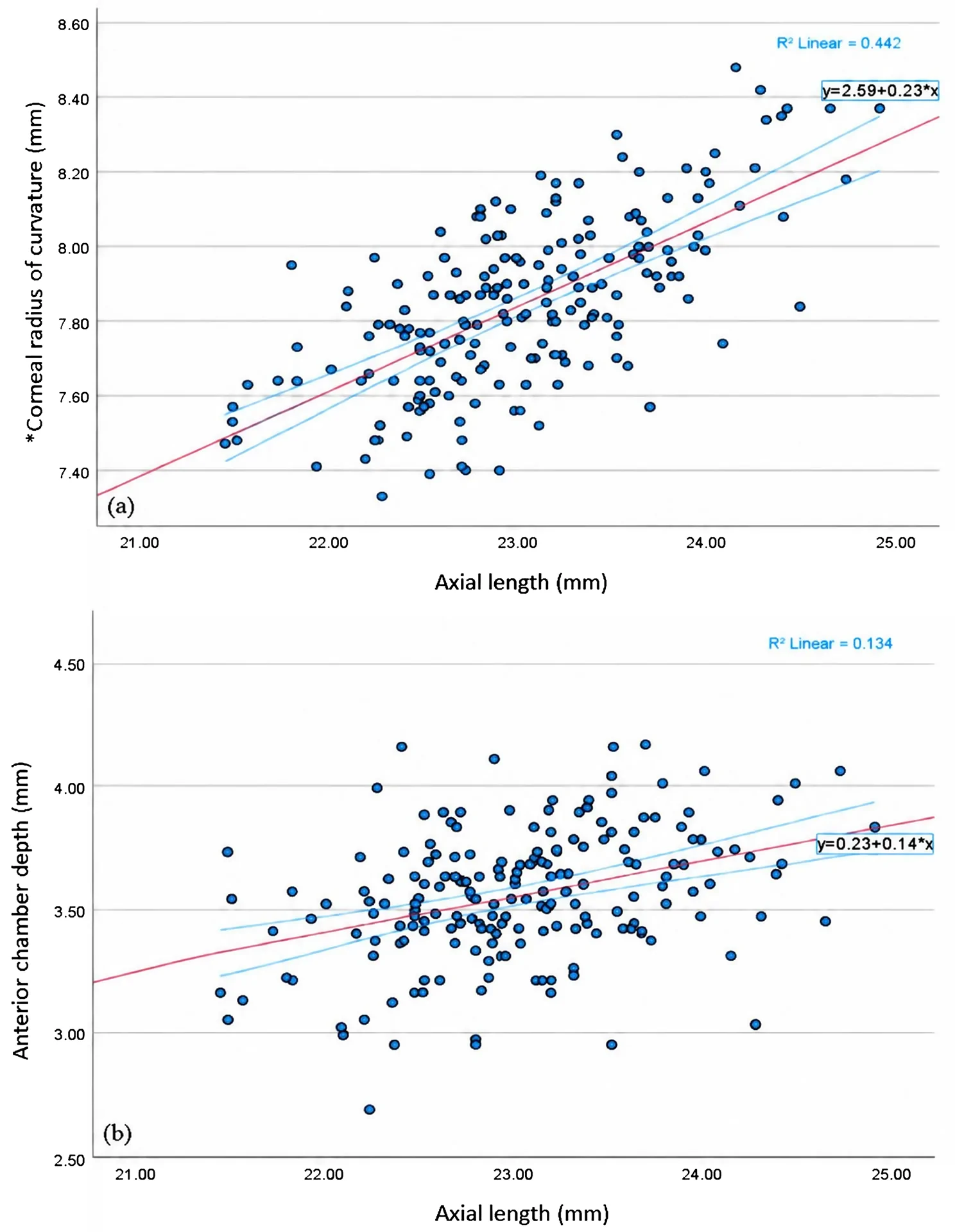
Scatter plot diagram showing correlation between (**a**) Axial length and Corneal radius of curvature, (**b**) Axial length and Anterior chamber depth among emmetropic Nepalese children. The red line indicates the line of best fit (regression line) and the two blue lines above and below indicates confidence interval bands (95%confidence interval)

Pearson’s correlation coefficient (r) was used to assess linear relationships between axial length and corneal radius of curvature, and between axial length and anterior chamber depth. Axial length demonstrated a strong positive correlation with the corneal radius of curvature **(r = 0.70, R**^**2**^ **=0.44**,** p < 0.001)**, indicating longer axial length corresponds to a flatter cornea while shorter axial length corresponds to a steeper cornea as shown in Fig. 3(a). Similarly, a moderate positive correlation was observed between axial length and anterior chamber depth **(r = 0., R**^**2**^ **=0.13**,** p < 0.001)**, indicating that eyes with longer axial lengths tended to have deeper anterior chambers, while shorter axial lengths tended to have shallow anterior chamber depth as shown in Fig. 3(b).

### Correlation between axial length-to-corneal radius ratio (AXL/CR) and spherical equivalent refraction (SER)

**Fig. 4:**
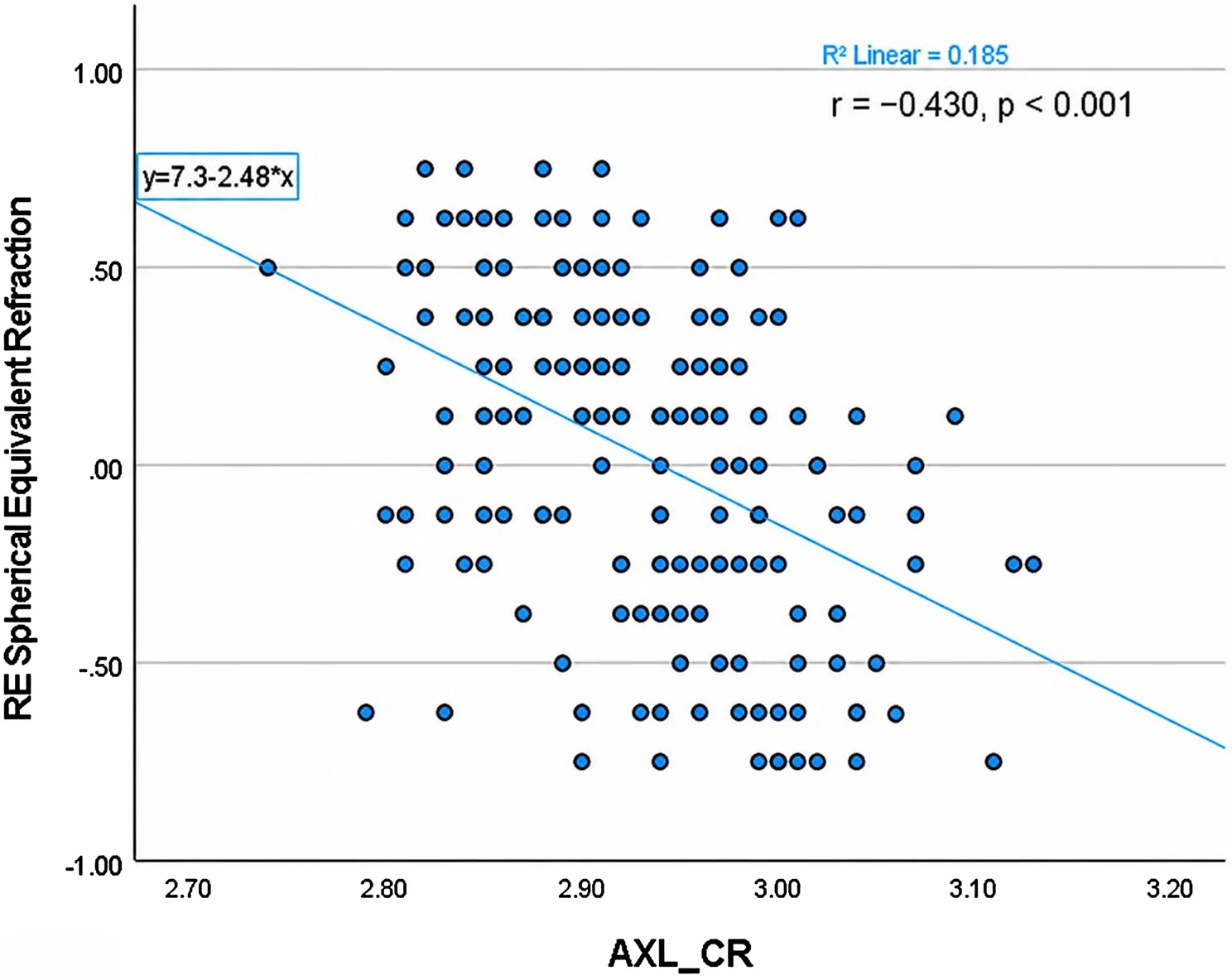
Scatter plot diagram showing correlation between axial length-to corneal radius and spherical equivalent refraction among emmetropic Nepalese children

Pearson’s correlation was used to assess relationship between axial length-to-corneal radius of curvature ratio and spherical equivalent refraction. A statistically significant moderate negative correlation was observed between axial length-to-corneal radius ratio and spherical equivalent refraction **(r = − 0.43**,** R**^**2**^**= 0.185**,** p < 0.001)**, indicating that higher AXL/CR values were associated with more negative SER as shown in Fig. 4.

## Discussion

This study documents ocular biometric parameters-including corneal radius of curvature, axial length, anterior chamber depth, lens thickness, pachymetry, pupil diameter, white-to-white diameter and axial length/corneal radius of curvature ratio, as well as corneal topographical parameters such as corneal eccentricity index, average central corneal power, and Fourier indices in a hospital-based sample of 190 emmetropic Nepalese children aged 6–18 years using OA-2000 optical biometer.

Based on the overall mean values of ocular biometric components reported in previous comparable studies, some of the ocular biometric parameters were reported to differ across similar age group [3, 13]. These discrepancies observed between studies may partly reflect variations in sample size and age-group distribution, in addition to differences in population characteristics and measurement techniques, particularly the use of ultrasound contact biometry for assessing ocular biometric parameters which includes measurement of axial length only up to inner limiting membrane and underestimation of anterior chamber depth due to corneal indentation [7, 14]. This highlights the importance for accurate measurement of these parameters which is possible with the advent of optical biometer that measures axial length extending to the retinal pigment epithelium and eliminates corneal indentation during anterior chamber assessment, thereby overcoming the limitations of ultrasound contact biometry.

The mean corneal curvature values observed in our study were comparable to those reported by He et al. [13] among Chinese children, whereas slightly lower values were reported by Hashemi et al. [3, 15] among Iranian children. These inter-study variations may reflect differences in ethnicity and genetic background among Iranian and Nepalese children. Previous studies [16, 17] have reported shorter axial length as compared to our study. However, these comparisons should be interpreted cautiously because differences in age distribution and refractive status between study populations contributes to variations in axial length. In particular, comparisons with predominantly myopic Asian populations may not be directly applicable to our emmetropic sample, as myopic eyes generally have longer axial lengths than emmetropic eyes. Therefore, comparisons with studies involving children of similar age and refractive status provide a more appropriate context for interpreting our findings. In this context, Hashemi et al. and He et al. reported higher mean axial length values among Iranian and Chinese children respectively, despite having comparable age ranges and refractive status [3, 13]. Other factors, including ethnicity, lifestyle, geographical location, environmental exposure, and near-work activities, may also contribute to differences in ocular biometric parameters [5, 18, 19]. 

The mean anterior chamber depth observed in our study was slightly higher than that reported by Garner et al. [6] among Tibetan children. This discrepancy may partly reflect differences in refractive status between the study populations, as myopic eyes generally have a deeper anterior chamber than emmetropic and hyperopic eyes. In addition, methodological differences, particularly the use of contact ultrasound biometry for anterior chamber depth assessment, may have contributed to the observed variation. Previous studies [16, 20, 21] have demonstrated an increase in anterior chamber depth with age in both genders, which aligns with our findings. Similarly, evidences from prior studies also demonstrated the similar mean lens thickness values compared to those observed in our study [3, 6, 17]. However, the mean pachymetry values in our study were lower than those reported in previous studies [3, 17, 22, 23]. Due to variations in ethnic groups and environmental factors such as altitude, UV exposure, results in slight variations in pachymetry values [24]. 

Many prior studies [25, 26] have reported lower mean pupil diameter value when compared to the findings of this study. This may be due to illumination condition of different measurements devices as well as ethnicity affects the pupil diameter. Also, the mean corneal diameter value in our study was consistent with the findings of prior studies [27–30]. This supports the validity of the OA-2000 measurements in our study population. Furthermore, Foo et al. reported the mean value of AXL/CR ratio to be 2.91 which aligns with our study [31]. A higher AXL/CR ratio (i.e., > 3.0) has been associated with a shift toward myopic development, incorporating axial length and corneal curvature into myopic screening may contribute to identify children at increased risk of myopia at an early age [7]. 

Our findings were consistent with coordinated ocular growth during emmetropization, involving passive, age-related changes in corneal curvature and anterior segment dimensions alongside active, visually guided axial growth regulation in response to retinal image quality. However, the underlying active regulatory mechanisms were not directly assessed in this study. A strong positive correlation between axial length and average corneal radius (*r* = 0.70), consistent with Hashemi et al. [3], suggests that corneal flattening partly compensates for axial elongation supporting the idea of emmetropization. In contrast, Park et al. reported a negative correlation (*r*= -0.44) [32] indicating an association between axial elongation and corneal steepening. This discrepancy may be attributed to disruption of normal emmetropization process, ethnicity, refractive status and differences in ocular anatomical structures. Similarly, a moderate positive correlation between axial length and anterior chamber depth (*r* = 0.40), consistent with previous studies [3, 32], indicates coordinated changes in anterior segment dimensions [21]. Disruption of this coordination, particularly disproportionate axial elongation, may contribute to refractive error. Additionally, He et al. reported a negative correlation between axial length-to-corneal radius ratio and SER (*r*= -0.25) which was lower than our findings, supporting the relevance of AL/CR ratio to refractive development [33]. 

Our analysis revealed a statistically significant age-related differences only for axial length and anterior chamber depth (*p* < 0.05). Similarly, gender differences were statistically significant for most of the parameters, (e.g., < 0.1 mm for corneal curvature and ACD, < 0.05 mm for LT) suggests that the clinical relevance of these findings is limited to the larger differences observed in AXL (~ 0.4 mm) and WTW (~ 0.19 mm), suggesting that males exhibited flatter corneas, longer axial length, deeper anterior chamber depth and larger white-to-white corneal diameter compared to females, consistent with previous studies [5, 7, 18, 20, 29]. These differences may be attributed to larger body size, body mass and ocular dimensions in males compared to that of females but these anthropometric measurements were not measured in the present study. Furthermore, no statistically significant differences for lens thickness were observed across gender and age groups, suggesting relatively uniform lens growth with increasing age, consistent with the findings reported by Rauscher et al. [17].

Most existing literature on corneal topography has been conducted in vernal kerato- conjunctivitis, blepharo-conjunctivitis and keratoconus globally [34–36]. No previous studies have reported normative corneal topographical screening parameters using an optical biometer among emmetropic Nepalese children. The mean CEI (0.39 ± 0.19), corneal asymmetry (0.44 ± 0.16 D), and higher order aberration in 3 mm corneal zone (0.18 ± 0.04 D) in this study provides useful reference data for assessing corneal irregularity in children. Values substantially outside this range, may warrant further evaluation with corneal tomography for suspected early ectatic changes, although these values should not be considered diagnostic thresholds for keratoconus. In addition, characterization of the corneal astigmatic component is important when considering the refractive outcome following pediatric cataract surgery, pediatric IOL power assessment and selected toric IOL planning. In this study, mean regular corneal astigmatism observed (0.38 ± 0.19 D) provides a useful normative reference and may assist in identifying children with clinically meaningful astigmatic deviations. To our knowledge, this is the first report of normative corneal topographical screening parameters (CEI, ACCP, Fourier indices) of both eyes in emmetropic Nepalese children using an optical biometer. These data fill a critical gap, serving as a reference for early diagnosis of corneal disorders (e.g., keratoconus), refractive surgery planning, and pediatric IOL power calculation.

The hospital-based recruitment may also introduce selection bias, and the study population may not fully represent Nepalese children from rural settings, different socioeconomic backgrounds, or differing lifestyle and environmental exposures. The moderate sample size and unequal distribution across age groups, particularly the smaller number of participants aged ≥ 16 years, may further limit generalizability. Consequently, the use of SER alone in categorizing refractive error may result in the inclusion of eyes with clinically significant astigmatism within the emmetropic group and may potentially overestimate the proportion of children classified as emmetropic. This limitation should be considered when interpreting the biometric characteristics of the study population. Nevertheless, these findings provide a valuable baseline source for future research and clinical applications in pediatric eye health.

## Conclusion

Significant gender-related differences were observed for most ocular biometric parameters, however, the magnitude of these differences varied; with larger differences observed for axial length and white-to-white diameter. The observed correlations between axial length, corneal curvature, and anterior chamber depth underscore their interdependent roles in emmetropization, reflecting compensatory mechanisms that support normal refractive development. These findings enhance understanding of pediatric ocular growth in Nepalese children and offer a normative reference for myopia control strategies, intraocular lens power calculation, and clinical evaluation of pediatric eye health.

## Data Availability

The data generated or analyzed in this study are available from the corresponding author upon reasonable request.
